# Jagged1-induced Notch activation contributes to the acquisition of bortezomib resistance in myeloma cells

**DOI:** 10.1038/s41408-017-0001-3

**Published:** 2017-12-15

**Authors:** Yukari Muguruma, Takashi Yahata, Takayuki Warita, Katsuto Hozumi, Yoshihiko Nakamura, Rikio Suzuki, Mamoru Ito, Kiyoshi Ando

**Affiliations:** 1 Center for Regenerative Medicine, Tokai University School of Medicine, Isehara, Japan; 2 Department of Hematology and Oncology, Tokai University School of Medicine, 143 Shimokasuya Isehara, Kanagawa 259-1193 Isehara, Japan; 3 Department of Immunology, Tokai University School of Medicine, Isehara, Japan; 4 Central Institute for Experimental Animals, Kawasaki, Japan

Multiple myeloma (MM) is a hematologic malignancy characterized by the proliferation of malignant plasma cells in the bone marrow (BM)^1^. Despite recent advances in therapy^2^, MM remains incurable, largely due to the emergence and maintenance of drug-resistant myeloma cells^3^, whose interactions within the BM microenvironment, the BM myeloma niche, are critical^4^. Because Notch signaling, a pathway activated only via cell-cell contacts, has been implicated as an integral part of the onset and progression of MM^5^, we investigated how Notch activation in myeloma cells, specifically through interactions with ligand-expressing niche cells, affects the pathophysiology of MM.

We first demonstrated the presence of at least one Notch receptor in 10 arbitrarily selected MM cell lines (Supplementary Figure 1a and b). The presence of these receptors indicates that Notch signaling can be activated and is functional in human myeloma cells. Of the two Notch ligands examined, Jagged1, but not Jagged2, is known to be abundantly expressed in many types of cells in the BM myeloma niche^6, 7^. Therefore, we investigated the effects of niche-induced Jagged1-Notch activation on myeloma cells. To distinguish niche-induced Notch activation from the homotypic activation of Notch in myeloma cells, in which Notch and its ligands can be simultaneously expressed, we selected five MM cell lines that expressed Jagged1 weakly or not at all for analyses. Contrary to previous reports^8^, the presence of immobilized human Jagged1 did not significantly alter the proliferation of any of the five MM cell lines in vitro (Supplementary Figure 1c). To further investigate the roles of niche-induced Notch signaling, we established a clinically relevant animal model of human MM that would allow us to examine myeloma cell interactions within the BM niche (Supplementary Figure 1d), where malignant plasma cells primarily proliferate in patients with MM. The engraftment of human MM cells in the BM did not increase even when U266 cells were transplanted into non-obese diabetic/severe combined immunodeficient/IL2Rγnull (NOG) mice expressing human Jagged1 in osteoblasts (NOGJ), a cell type that constitutes the BM myeloma niche (Supplementary Figure 1e). The results indicate that niche-induced Jagged1-Notch signaling is not specifically associated with the proliferation of myeloma cells.

We then assessed whether niche-induced Jagged1-Notch activation affects the sensitivity of myeloma cells to drugs used clinically to treat MM patients. In co-culture experiments, transgenic expression of human *Jagged1* in stromal cells (ST2J) increased the survival of U266 cells only when the cells were exposed to bortezomib (BTZ), which coincided with the upregulation of human *Hey1* and *Hes1* expression in culture (Supplementary Figure 2a-c), indicating a role of Notch signaling in BTZ resistance. Five other MM cell lines similarly demonstrated resistance to BTZ in the presence of ST2J cells (Supplementary Figure 2d). As expected, cells cultured on immobilized human Jagged1 demonstrated significant resistance to BTZ treatment (Fig. 1a) but not to melphalan treatment (Supplementary Figure 2e). In addition, a marked upregulation in the expression of *Hey1* and *Hes1* was detected in cells that survived BTZ treatment (Fig. 1b), confirming that the observed resistance to BTZ is indeed a direct result of Jagged1-induced Notch activation in myeloma cells.

**Fig. 1:**
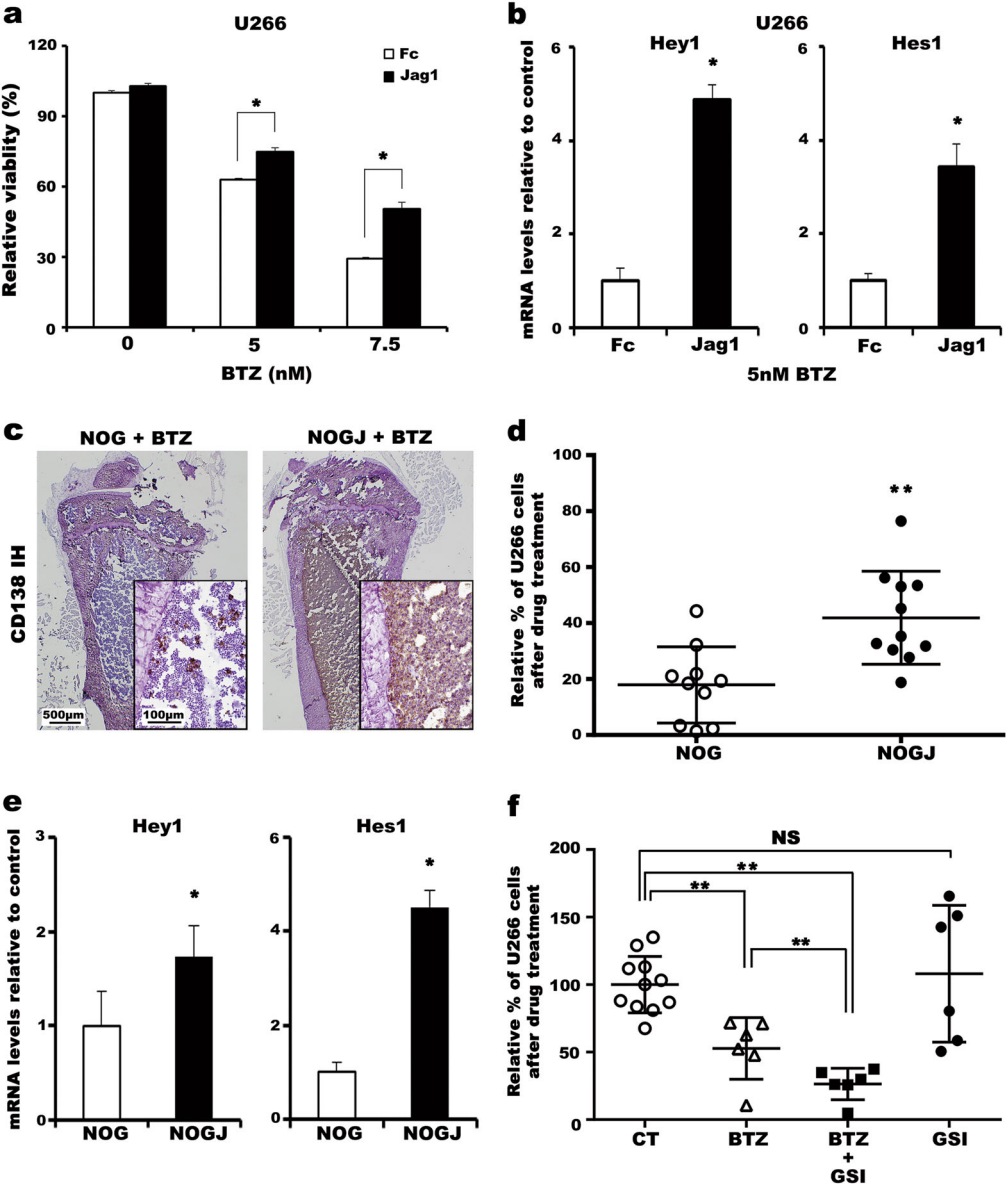
Jagged1-induced Notch activation augmented human myeloma cell survival against BTZ treatment in vitro and in vivo **a** U266 cells (2 × 10^4^ cells/well) were cultured in medium containing the indicated concentrations of BTZ in the presence of immobilized recombinant human Jagged1-Fc chimera protein or the control Fc fragment. Analyses were performed at least in triplicate wells. Representative results from nine independent experiments are shown. Bars represent % ATP activity relative to the control culture with 0 nM BTZ. **b** The expression of *Hey1* and *Hes1* in surviving cells after treatment with 5 nM BTZ was measured by Taqman-PCR. Bars represent the relative mRNA expression compared with the control culture. Analyses were performed in quadruplicate wells. Representative results from two independent experiments are shown. The 18S rRNA gene was used as a reference gene. **c** BM sections obtained from NOG or NOGJ mice were stained with an antibody to human CD138, a clinically established marker for the identification of malignant plasma cells. Brown spots represent human myeloma cells expressing CD138. Representative images from five independent experiments are shown. Inserts are higher magnification images. **d** Survival of human myeloma cells in the BM of NOG and NOGJ mice after BTZ treatment. The relative survival of myeloma cells was calculated by dividing the percentage of myeloma cells remaining after BTZ treatment by the percentage of myeloma cells in the BM of saline-treated control mice. Each circle represents the calculated myeloma cell survival value. Pooled data from five independent experiments are shown (*n* = 11). **e** The expression of *Hey1* and *Hes1* in surviving cells after BTZ treatment in vivo was measured by Taqman-PCR. Bars represent the relative mRNA expression compared with the control mice. Analyses were performed in quadruplicate wells. Representative results from four independent experiments are shown. Human β-actin was used as a reference gene. **f** Survival of human myeloma cells after drug treatment. The relative survival of myeloma cells was calculated by dividing the percentage of myeloma cells remaining after drug treatment by the percentage of myeloma cells in the BM of control mice. Each circle represents the calculated value for an individual mouse. Pooled data from four independent experiments are shown (*n* = 11 for the control group, others *n* = 6). **a**, **b**, and **d**–**f** Error bars, mean ± SD; *P < 0.05; **P < 0.01; *NS*, not significant

We therefore tested the in vivo effects of niche-induced Jagged1-Notch activation on sensitivity to myeloma therapy by treating human MM mice with BTZ (Supplementary Figure 3a). The BM of NOGJ recipients was infused with human myeloma cells, in contrast to the BM of NOG mice, in which myeloma cells were effectively cleared by treatment with the same dose of BTZ, as determined by qualitative immunohistochemical and quantitative flow cytometric analyses for CD138 expression (Figs. 1c and d). The survival of myeloma cells in BTZ-treated NOGJ mice was enhanced approximately two-fold compared with that of NOG mice (41.91 ± 16.63% in NOGJ mice and 18.93 ± 12.60% in NOG mice). Consistent with our earlier observations, the expression of both *Hey1* and *Hes1* was significantly upregulated in human myeloma cells that survived BTZ treatment in the NOGJ environment (Fig. 1e), supporting our hypothesis that niche-induced activation of Jagged1-Notch signaling is critical in the acquisition of resistance to BTZ. We therefore examined the effect of Notch inhibition in human MM mice (Supplementary Figure 3b). Administration of a suboptimal dose of BTZ reduced the survival of myeloma cells by approximately 50% (52.7 ± 22.8%). The combination of BTZ and a γ-secretase inhibitor (GSI)further reduced the survival of myeloma cells by half (26.4 ± 11.6%, Fig. 1f), which is nearly equivalent to the extent observed in the earlier experiment using a higher dose of BTZ, even though injection of a GSI alone did not significantly alter the survival of human myeloma cells. Taken together, these results experimentally confirmed that the activation of Notch in myeloma cells through an interaction with Jagged1-expressing niche cells is responsible for the acquisition of BTZ resistance.

To mechanistically determine how niche-induced Jagged1-Notch signaling protects human myeloma cells from BTZ treatment, we examined the expression and activation of myristoylated alanine-rich C-kinase substrate (MARCKS) in BTZ-treated cells. MARCKS is a substrate of protein kinase C (PKC) and has been found to be overexpressed in several cancers, including MM^9, 10^. However, at present, how MARCKS is initially activated remains to be determined. When U266 cells were treated with BTZ or melphalan in vitro, MARCKS expression and activation were significantly downregulated, indicating that MARCKS is involved in myeloma cell survival (Fig. 2a). Interestingly, when cells were cultured on immobilized human Jagged1, MARCKS phosphorylation was maintained in cells treated with BTZ but not in cells treated with melphalan, the first demonstration of a link between Notch activation and a PKC pathway in acquiring BTZ resistance. The involvement of MARCKS activation in Jagged1-induced acquisition of BTZ resistance was confirmed in MM1S cells (Supplementary Figure 4). We therefore determined whether inhibitors of PKC signaling can counteract the Jagged1-induced survival of myeloma cells against BTZ. As expected, a combination of BTZ and PKC inhibitors attenuated survival of myeloma cells in vitro. The addition of GF109203X, a pan-PKC inhibitor, effectively reduced the viability of myeloma cells and, at the same time, neutralized the Jagged1-induced enhanced survival of the myeloma cells (Fig. 2b). By contrast, the effects of Gö6976, an inhibitor of PKCα and β, and enzastaurin, a specific inhibitor for PKCβ, were barely significant, and they did not affect the Jagged1-Notch-mediated enhanced survival of myeloma cells against BTZ. Consistent with this finding, GF109203X significantly downregulated MARCKS activation and nullified the Jagged1-induced maintenance of phosphorylated MARCKS (Fig. 2c). Meanwhile, Jagged1-mediated sustained phosphorylation of MARCKS still occurred when other PKC inhibitors were used, implying that the PKCs involved in BTZ resistance may be unique to individual patients given the heterogeneity of the pathophysiology among MM patients. The addition of a GSI to the culture abolished the sustained phosphorylation of MARCKS in the presence of Jagged1 (Fig. 2d), supporting the link between Notch activation and PKC signaling. Finally, the importance of persistent MARCKS activation in the acquisition of BTZ resistance was confirmed using a MARCKS gene-silenced MM cell line (siMARCKS cells) both in vitro and in vivo (Supplementary Figure 3c-e). MARCKS knockdown effectively prevented the survival of myeloma cells in vivo, even though it did not affect the initial engraftment of myeloma cells in the BM (Fig. 2e). Altogether, our results indicate that niche-induced Jagged1-Notch signaling activates PKC, which then phosphorylates MARCKS and contributes to the survival of myeloma cells.

**Fig. 2:**
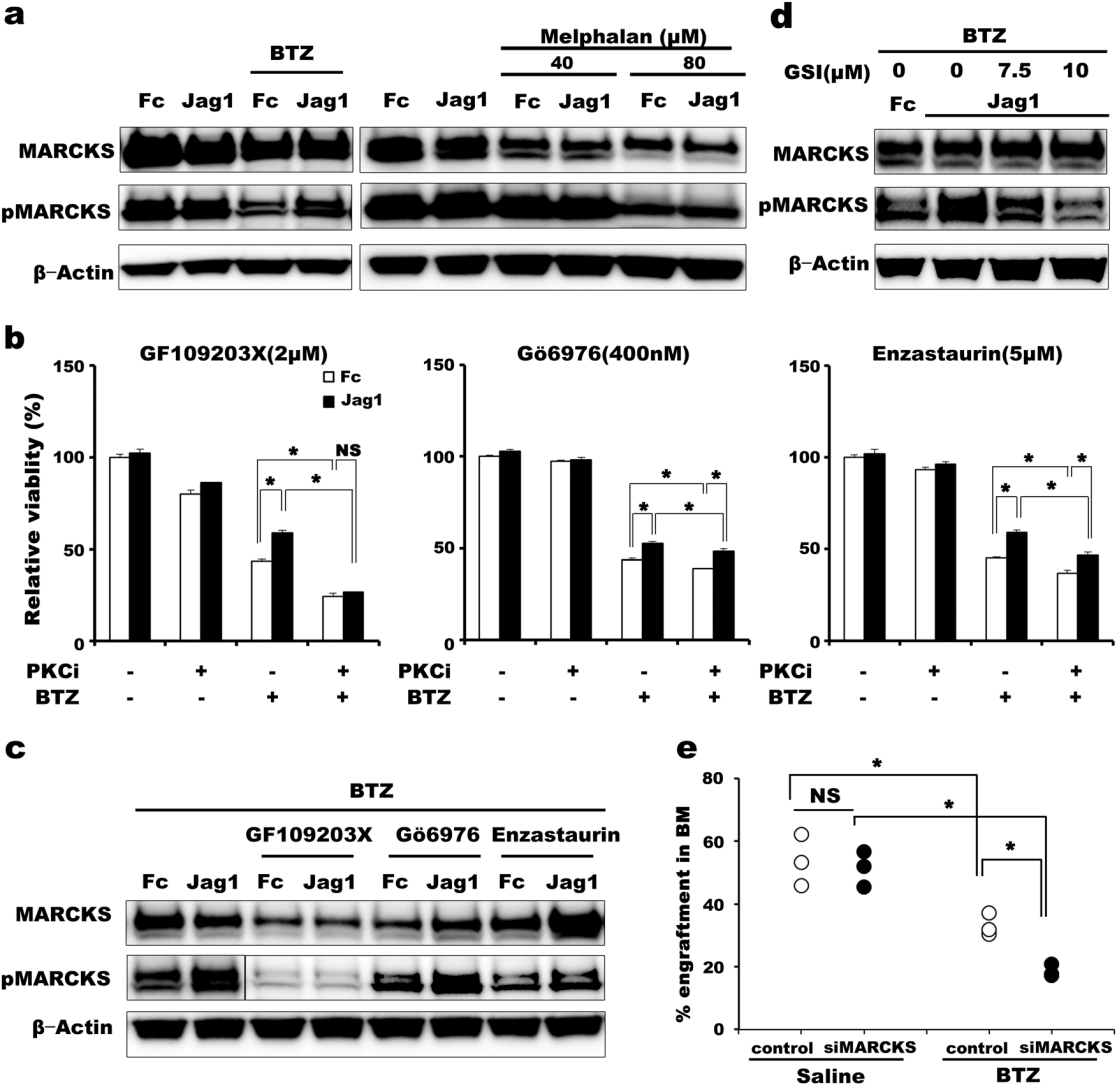
PKC-MARCKS signaling was involved in Notch-mediated survival of human myeloma cells **a** MARCKS expression and activation were reduced in the presence of BTZ and melphalan. Jagged1-Notch signaling maintained MARCKS phosphorylation in U266 cells only when cells were exposed to BTZ. Representative images of two independent experiments are shown. **b** U266 cells (2 × 10^4^ cells/well) were cultured in medium containing 7.5 nM BTZ combined with GF109203X, Gö6976, enzastaurin, or DMSO in the presence of immobilized recombinant human Jagged1-Fc chimera protein or control Fc fragment. Bars represent % ATP activity relative to the Fc control well cultured with the appropriate concentration of DMSO. Analyses were performed at least in triplicate wells. Representative results from five to seven independent experiments are shown. Error bars, mean ± SD. **c** Addition of PKC inhibitors significantly inhibited MARCKS phosphorylation. Extended exposure was needed to achieve band densities comparative to those without PKC inhibitors. In the pMARCKS lane, the results obtained after different exposure times are separated by a black line. Use of GF109203X nullified the maintenance of MARCKS phosphorylation in the presence of Jagged1. **d** Inhibition of Notch signaling with a GSI abolished the Jagged1-mediated sustained MARCKS activation in a dose-dependent manner. **a**, **c**, and **d** MARCKS expression and activation were analyzed by western blotting. Representative images of four (**a**) and two (**c**,** d**) independent experiments are shown. **e** Survival of human myeloma cells after drug treatment. The presence of siMARCKS or control U266 cells in the BM was measured by flow cytometry. Each symbol represents the % of human myeloma cells in the BM. **b**, **e** *P < 0.05; *NS*, not significant

This study identifies the unique role of niche-induced Notch activation in the pathogenesis of MM. While niche-induced Jagged1-Notch activation is responsible for the acquisition of BTZ resistance, it does not appear to be involved in either melphalan resistance or myeloma cell proliferation. By focusing on the niche-induced activation of Notch signaling, we successfully demonstrated that Jagged1, which is expressed in niche cells, activates a Notch-PKC pathway in myeloma cells and that, as part of this pathway, MARCKS plays an important role in the emergence of drug-resistant myeloma cells. In addition, this study presents experimental evidence that modulation of PKC signaling is an effective strategy for counteracting the emergence of drug-resistant cells induced by the activation of Notch signaling.

Because of its causative association with many types of disease, including cancer^11^, Notch signaling has been an attractive therapeutic target. However, severe adverse reactions to pharmacological agents that inhibit Notch signaling preclude the development of drugs that can be used clinically to treat patients^12, 13^. In this study, the addition of a pan-PKC inhibitor neutralized the Jagged1-induced acquisition of BTZ resistance in vitro, which provides promising evidence for the use of PKC inhibitors as Notch signaling modulators in MM. Because MARCKS, a molecule important for myeloma cell survival, has also been shown to be involved in the adhesion and metastatic invasion of tumor cells in solid tumors^14, 15^, niche-induced Jagged1-Notch activation may also participate in the localization and migration of malignant plasma cells into and from the BM milieu to extramedullary proliferation sites, processes that are critically involved in the progression of MM. This study provides a rationale for the PKC-MARCKS pathway as a druggable target in refractory MM.

## Electronic supplementary material

Supplementary Information

Supplementary Figures

## References

[1] Palumbo A, Anderson K (2011). Multiple myeloma. N. Engl. J. Med..

[2] Moreau P (2012). Proteasome inhibitors in multiple myeloma: 10 years later. Blood.

[3] Abdi J, Chen G, Chang H (2013). Drug resistance in multiple myeloma: latest findings and new concepts on molecular mechanisms. Oncotarget.

[4] Podar K, Chauhan D, Anderson KC (2009). Bone marrow microenvironment and the identification of new targets for myeloma therapy. Leukemia.

[5] Colombo M (2013). Notch-directed microenvironment reprogramming in myeloma: a single path to multiple outcomes. Leukemia.

[6] Li L (1998). The human homolog of rat Jagged1 expressed by marrow stroma inhibits differentiation of 32D cells through interaction with Notch1. Immunity.

[7] High FA (2008). Endothelial expression of the Notch ligand Jagged1 is required for vascular smooth muscle development. Proc. Natl Acad. Sci. U.S.A..

[8] Jundt F (2004). Jagged1-induced Notch signaling drives proliferation of multiple myeloma cells. Blood.

[9] Yang Y (2015). Targeting phospho-MARCKS overcomes drug-resistance and induces antitumor activity in preclinical models of multiple myeloma. Leukemia.

[10] Micallef J (2010). Applying mass spectrometry based proteomic technology to advance the understanding of multiple myeloma. J. Hematol. Oncol..

[11] Lobry C, Oh P, Aifantis I (2011). Oncogenic and tumor suppressor functions of Notch in cancer: it’s NOTCH what you think. J. Exp. Med..

[12] van Es JH (2005). Notch/gamma-secretase inhibition turns proliferative cells in intestinal crypts and adenomas into goblet cells. Nature.

[13] Wong GT (2004). Chronic treatment with the gamma-secretase inhibitor LY-411,575 inhibits beta-amyloid peptide production and alters lymphopoiesis and intestinal cell differentiation. J. Biol. Chem..

[14] Estrada-Bernal A, Gatlin JC, Sunpaweravong S, Pfenninger KH (2009). Dynamic adhesions and MARCKS in melanoma cells. J. Cell. Sci..

[15] Yang Z (2016). MARCKS contributes to stromal cancer-associated fibroblast activation and facilitates ovarian cancer metastasis. Oncotarget.

