# Supplementary material for: Jagged1-induced Notch activation contributes to the acquisition of bortezomib resistance in myeloma cells
Source: Blood Cancer J. 2017 Dec 15;7(12):650. doi: 10.1038/s41408-017-0001-3 (PMC5802593; doi:10.1038/s41408-017-0001-3)
Supplement: Supplementary file 1 — Supplementary Information [file 41408_2017_1_MOESM1_ESM.pdf]

Supplementary Information

Supplementary Methods

Materials and antibodies

The recombinant human Jagged1-Fc chimera was obtained from R&D Systems (Minneapolis, MN), the human IgG Fc fragment was from Jackson ImmunoResearch (West Grove, PA), and recombinant protein G was from BioVision (Milpitas, CA). BTZ was purchased from Janssen Pharmaceutical (Tokyo, Japan), and GSI XII was from Calbiochem (San Diego, CA). Melphalan and thalidomide were purchased from Sigma-Aldrich (Saint Louis, MO). The PKC inhibitor Gö6976 was obtained from Cell Signaling Technology (CST, Danvers, MA), and GF109203X and enzastaurin (LY317615) were from Selleck Chemicals (Houston, TX). Antibodies against the following proteins were used to detect protein expression by western blotting: Notch1, Notch2, Jagged2, MARCKS, and phospho-MARCKS (CST); Jagged1 (Santa Cruz Biotechnology, Santa Cruz, CA); and β–actin and GAPDH (Sigma-Aldrich). For flow cytometric analyses of human myeloma cells, FITC-conjugated anti-murine CD45 and Ter119 (Biolegend, San Diego, CA) and APC-conjugated anti-human CD138 or PE-conjugated anti-human CD38 (both from BD PharMingen, San Jose, CA) were used. To detect human myeloma cells in immunohistochemical analyses, an anti-human CD138 antibody (mouse monoclonal, clone MI15, DAKO Cytomation, Denmark) was used. shRNA lentiviral particles for human MARCKS and control shRNA were purchased from Santa Cruz Biotechnology.

Cell lines and cell culture

The human multiple myeloma (MM) cell lines RPMI8226 (JCRB0034), delta-47 (JCRB1344), IM9 (IFO50025), KMM-1 (JCRB1180), KMS11 (JCRB1179), and KMS12-BM (JCRB0429) were purchased from the Japanese Collection of Research Bioresources cell bank (Osaka, Japan), NCI-H929 (CRL-9068) cells were purchased from the American Type Culture Collection (Manassas, VA), and ARH-77 (ACC512) cells were obtained from Deutsche Sammlung von Mikroorganismen und Zellkulturen GmbH (Braunshweig, Germany). U266 and OPM-2 cells were kindly provided by Dr. Shinsuke Iida (Nagoya City University, Aichi, Japan). All human cell lines were propagated in RPMI 1640 medium supplemented with 10% FBS. Cells in the logarithmic growth phase were frozen in aliquots and stored in liquid nitrogen until use. The cells were cultured between 15 and 20 days before being used in experiments. A murine BM stromal cell line, ST2, was lentivirally infected with human *Jagged1* cDNA cloned into a GCDNsap vector[1, 2] that expresses a truncated human nerve growth factor receptor as a surrogate expression marker and used for co-culture experiments described below.

Preparation of Jagged1 coated plates

Jagged1-coated plates were prepared as described before[3]. Briefly, plates were incubated with 50 μg/ml protein G in phosphate-buffered saline (PBS) overnight, washed three times with PBS, and blocked with 1 %BSA in PBS

for 4 hours at 4 ℃. After being washed with PBS, plates were incubated with 5 μg/ml of recombinant human Jagged1-Fc chimera or Fc fragment of human IgG overnight at 4 ℃.

Proliferation and cell viability assays

The proliferation and viability of MM cell lines were measured using the CellTiter-Glo® Luminescent Cell Viability Assay reagent (Promega, Madison, WI). MM cell lines were plated in a 96-well flat-bottomed culture tray prepared as described above at the concentration predetermined for individual cell lines that allowed logarithmic growth of cells during a 48-hour incubation period (0.5-3X10^4 cells/well). For cell viability assay, BTZ dissolved in saline, PKC inhibitors dissolved in DMSO, or the combination of both drugs were added at the indicated concentrations. The same volume of DMSO was added to the control wells. Alternatively, myeloma cells were cultured on a pre-established monolayer of irradiated (17 Gy) human *Jagged1*-transfected (ST2J) or mock-transfected ST2 cells in the presence of BTZ, melphalan dissolved in acid-ethanol, thalidomide dissolved in DMSO, or the same volume of appropriate control solution.

RNA purification and gene expression analysis

Total RNA was purified from cultured cells or tissue samples obtained from human myeloma mice using Isogen II solution (Nippon Gene Co, Toyama, Japan) according to the manufacturer’s instructions and reverse transcribed into cDNA. The expression of Notch signaling molecules was quantified

using appropriate Taqman® probes (Applied Biosystems, Foster City, CA). *hHes1*: Hs00172876, *hHey1*: Hs01114113, *hGAPDH*: Hs9999905, *hβ-actin*: Hs999903, *hNotch1*: Hs01062014, *hNotch2*: Hs01050702, *hJagged1*: Hs01070032, *mJagged1*: Mm00496902, *mJagged2*: Mm01325629, *18S rRNA*: Hs9999901. The relative amount of target gene expression was determined in reference to human *GAPDH*, human *β-actin*, or *18S rRNA*. A comparative threshold cycle (CT) was used to quantify transcripts. The value was calculated by the expression $2^{-\Delta CT}$.

Western blot analyses

Cells in logarithmic growth condition or cells treated with BTZ, a GSI, or the combination of BTZ and PKC inhibitors were harvested, washed with Tris-HCl pH7.5 containing a phosphatase inhibitor cocktail tablet (PhosSTOP from Roche Diagnostics GmbH, Germany), and lysed with RIPA buffer containing 1 mM PMSF (both from CST). Protein concentration was determined using a DC Protein Assay kit (Bio-Rad, Hercules, CA). Equal amounts of protein were loaded onto NuPAGE 3-8 % Tris-Acetate gel, 7 % Tris-Acetate gel, or 4-12 % Bis-Tris gel (all from Life Technologies, Carlsbad, CA), depending on the molecular weight of the proteins of interest, and transferred to PVDF membranes. Membranes were probed with the antibodies listed in the main text followed by the peroxidase-conjugated appropriate secondary antibodies. Signals were detected by chemoluminescence using ECL Prime detection system (GE Healthcare, UK).

Mouse model of human myeloma

Non-obese diabetic/severe combined immunodeficient/IL2Rγnull (NOG) and NOG mice that express human *Jagged1* under the control of a 2.3-kb osteoblast-specific promoter region of the murine α1(I) collagen promotor (NOGJ) were obtained from the Central Institute for Experimental Animals (Kawasaki, Japan)[4]. The mice were maintained in sterile microisolator cages in the animal facility of the Tokai University School of Medicine and were treated in accordance with the institutional guidelines. All animal experiments were approved by the Animal Care Committee of Tokai University. For experiments, 8- to 12-week-old mice were used. Data were compared with sex- and age-matched appropriate control mice. Sample size was limited by ethical considerations. No randomization or blinding was used to allocate experimental groups and no animals were excluded from analysis. The mice were given an intravenous injection of 0.5-1.5X10^6 MM cells in the retro-orbital plexus in 100 μl of PBS and were humanely killed 5 to 6 weeks after transplantation. BM cells and bones were collected and used for analyses.

Drug administration

Three weeks after the transplantation of myeloma cells, the mice were assigned to the following 4 treatment groups. The BTZ group received 0.35 mg/kg, 0.4 mg/kg, or 0.5 mg/kg BTZ dissolved in saline, concentrations specifically determined for the purpose of the experiments, three times a

week for two to three weeks. The GSI group received 7.5 mg/kg GSI dissolved in 10% DMSO 5 times a week for two weeks starting at 4 weeks after transplantation. The combination group received both drugs, and the control group received the same volume of saline and DMSO.

Generation of MARCKS knockdown MM cells

MARCKS gene silencing was accomplished using shRNA lentiviral particles according to the manufacturer's instructions.

Histological analyses

Bones were excised, fixed overnight with 4 % paraformaldehyde in PBS at 4 ℃, infiltrated with sucrose, embedded in 4 % carboxymethyl cellulose, and frozen in liquid nitrogen. Frozen sections of undecalcified bones were prepared using Kawamoto's film method[5]. Immunohistochemical staining was performed as described[6, 7]. Slides were stained for anti-human CD138 (1:100) to detect human myeloma cells.

Flow cytometric analyses

For quantitative assessment of human myeloma cell engraftment in BM by flow cytometry, BM cells were stained with anti-murine CD45 and Ter119 as well as anti-human CD138 and CD38. Flow cytometry was performed using a FACS-Calibur instrument equipped with the Cell Quest software program (BD Bioscience). Dead cells stained with propidium iodide (PI) were excluded from data collection. The portion of the cell population that did not react with

anti-murine antibodies and stained positive for human antibodies was identified as human myeloma cells. The proportion of the designated cell fraction was determined by collecting 100,000 events. The data obtained from transgenic mice were compared with sex-matched littermate control mice.

Statistics

Statistical analyses were conducted with GraphPad Prism, version 5.0 (GraphPad Software). Student's two-tailed unpaired *t*-test was used to determine the significance of the difference between the mean values of two groups. One-way ANOVA followed by Tukey's post hoc test was used to compare the mean values among three or more independent groups. A normal distribution of the data was confirmed using the Kolmogorov-Smirnov test. The mean ± SD is presented in each graph. P values <.05 were considered significant. NS indicates not significant.

Supplementary Figure S1

(a) The expression of Notch and Notch ligands in 10 human MM cell lines was analyzed by western blotting. The expression of the transmembrane/intracellular regions of both Notch1 (120 kDa) and Notch2 (110 kDa) was detected in eight of the 10 MM cell lines, although the expression of both receptors was very weak in NCI-H929 (H929) cells. While Notch2 was expressed in the remaining two cell lines, Notch1 expression was detected only weakly in KMS11 cells and not at all in U266 cells. In addition to the major cleaved transmembrane region of the expected size for Notch1 and Notch2 (indicated by arrowheads), several bands of lower molecular weight can be seen. Jagged1 was expressed broadly at various intensities; it was strongly expressed in IM9, ARH-77, and KMS12-BM cells, expressed weakly but clearly in RPMI8226 (8226) and OPM-2 cells, and expressed faintly or not at all in U266, H929, KMM1, KMS11, and delta47 cells. However, Jagged2 expression was confined to five MM cell lines: 8226, H929, KMM1, KMS12, and delta47 cells. Single bands of the expected size are shown for Jagged1 and Jagged2 (both 150 kDa). The membranes were reprobed with GAPDH to confirm protein loading. Representative images from two independent experiments are shown.

(b) The expression of *Notch1*, *Notch2*, and *Jagged1* was measured by Taqman-PCR. Bars represent the relative mRNA expression of each gene compared with the expression of a reference gene in individual cell lines. Representative results from two independent experiments are shown.

(c) Human MM cell lines (0.5-3X10^4 cells/well) were cultured for 48 hours in

the presence of 5 μg/mL immobilized recombinant human Jagged1-Fc chimera protein or the control Fc fragment. Analyses were performed at least in triplicate wells. Representative results from three independent experiments are shown.

(d) Engraftment of human myeloma cells in the BM of NOG mice was determined by flow cytometric analyses 5 to 6 weeks after transplantation of $1X10^6$ cells (n=5 for each cell line). The numbers above each bar represent the mean engraftment values for each cell line. U266 was the only cell line that resulted in consistent and substantial engraftment in the BM of NOG mice, while other four cell lines formed subcutaneous tumors in recipient mice. Of note, the expression of Jagged2, a Notch ligand implicated in the self-renewal of myeloma cells[8, 9], did not facilitate the engraftment and proliferation of myeloma cells in the BM, arguing against its importance *in vivo*. (c and d) Error bars, mean ± SD.

(e) U266 cells (0.5-$1.5X10^6$ cells) were intravenously transplanted into NOG mice or NOG mice expressing human Jagged1 in osteoblast (NOGJ). Engraftment of U266 cells in the BM was measured by flow cytometry. Each symbol represents the % engraftment in the BM. Horizontal lines connect analytical pairs. Pooled data from six independent experiments are shown (n=10). NS, not significant.

Supplementary Figure S2

(a) The expression of Notch ligands in ST2 and ST2 cells expressing human *Jagged1* (ST2J) was analyzed by western blotting and Taqman-PCR. Bars

represent the relative mRNA expression using the mRNA value of murine *Jagged1* (mJag1) in ST2 cells as a control. The transgenic expression of human *Jagged1* augmented the expression of human *Jagged1* by approximately 100-folds and slightly increased murine *Jagged1* expression (4-folds) without affecting the expression of murine *Jagged2*.

(b) U266 cells ($3X10^4$ cells/well) were seeded onto a monolayer of ST2 cells or ST2 cells expressing human *Jagged1* (ST2J) in the presence of the indicated concentration of drugs or the control solvent. Analyses were performed at least in triplicate wells. Representative results from two to seven independent experiments are shown. Bars represent % ATP activity relative to the control culture.

(c) U266 cells were cultured with ST2 or ST2J cells in the presence or absence of BTZ. The expression of *Hey1* and *Hes1* in human myeloma cells was measured by Taqman-PCR using probes specific to human genes. Bars represent the relative mRNA expression. Analyses were performed in quadruplicate wells. Representative results from two independent experiments are shown. Human GAPDH was used as a reference gene.

(d) Human MM cell lines ($1\text{-}3X10^4$ cells/well) were seeded onto a monolayer of ST2 or ST2J cells in the presence of the indicated concentration of BTZ. Analyses were performed at least in triplicate wells. Representative results from two to three independent experiments are shown. Bars represent % ATP activity relative to the control culture.

(e) U266 cells ($2X10^4$ cells/well) were cultured in medium containing the indicated concentrations of melphalan in the presence of immobilized

recombinant human Jagged1-Fc chimera protein or the control Fc fragment. Analyses were performed at least in triplicate wells. Representative results from three independent experiments are shown. Bars represent % ATP activity relative to the control culture with the control solvent. (b-e) Error bars, mean ± SD; *, $P<0.05$; NS, not significant.

Supplementary Figure S3

(a) Mice that received U266 transplants were administered an optimal dose of BTZ[10] or an equivalent amount of saline 3 times a week for 3 weeks beginning at 3 weeks after transplantation. After the mice were euthanized, the BM was sectioned and stained for human CD138, or BM cells were analyzed in flow cytometry.

(b) NOG mice that received U266 transplants were treated with a suboptimal dose of BTZ alone, GSI alone, or a combination of the two drugs. Control mice received the same volume of saline and/or DMSO.

(c) MARCKS expression and activation were effectively downregulated in U266 cells transduced with MARCKS shRNA lentiviral particles (siMARCKS cells) as determined by Taqman-PCR and western blotting. Bars represent the relative mRNA expression compared with cells transduced with control shRNA lentiviral particles. Taqman-PCR analyses were performed in triplicate wells. The 18S rRNA gene was used as a reference gene. Representative results from two independent experiments are shown for both Taqman-PCR and western blotting analyses.

(d) siMARCKS or control U266 cells ($2X10^4$ cells/well) were cultured in

medium containing the indicated concentrations of BTZ in the presence of immobilized recombinant human Jagged1-Fc chimera protein or the control Fc fragment. Analyses were performed in triplicate wells. Representative results from four independent experiments are shown. Bars represent % ATP activity relative to the control culture with 0 nM BTZ. (c and d) Error bars, mean ± SD; *P<0.05

(e) NOG mice that received siMARCKS or control U266 transplants were treated with BTZ 3 times a week for 2 weeks.

Supplementary Figure S4

(a) The expression of Notch and Notch ligands in MM1S and MM1R cell lines were analyzed by western blotting and Taqman-PCR. Bars represent the relative mRNA expression. MM1S cells expressed relatively low levels of Jagged1. Distinct expression of Jagged1 and faint expression of Jagged2 (indicated by an arrowhead) were detected in MM1R cells.

(b) MM1S cell proliferation in the presence of immobilized recombinant human Jagged1-Fc chimera protein or the control Fc fragment was examined. Analyses were performed in triplicate wells. Representative results from three independent experiments are shown.

(c) MM1S cells ($3X10^4$ cells/well) were seeded onto a monolayer of ST2 or ST2J cells in the presence of the indicated concentration of BTZ. Analyses were performed in triplicate wells. Representative results from three independent experiments are shown. Bars represent % ATP activity relative to the control culture. (b and c) Error bars, mean ± SD; *, P<0.05; NS, not

significant.

(d) MARCKS expression and activation in MM1S cells cultured in the presence of BTZ were analyzed by western blotting. Jagged1-Notch signaling increased the expression of MARCKS and maintained its phosphorylation. Representative images of two independent experiments are shown.

Supplementary references

1. Nabekura T, Otsu M, Nagasawa T, Nakauchi H, Onodera M. Potent vaccine therapy with dendritic cells genetically modified by the gene-silencing-resistant retroviral vector GCDNsap. *Molecular therapy : the journal of the American Society of Gene Therapy* 2006 Feb; **13**(2): 301-309.

2. Suzuki A, Obi K, Urabe T, Hayakawa H, Yamada M, Kaneko S*, et al.* Feasibility of ex vivo gene therapy for neurological disorders using the new retroviral vector GCDNsap packaged in the vesicular stomatitis virus G protein. *Journal of neurochemistry* 2002 Aug; **82**(4): 953-960.

3. Wilhelmsson U, Faiz M, de Pablo Y, Sjoqvist M, Andersson D, Widestrand A*, et al.* Astrocytes negatively regulate neurogenesis through the Jagged1-mediated Notch pathway. *Stem cells* 2012 Oct; **30**(10): 2320-2329.

4. Negishi N, Suzuki D, Ito R, Irie N, Matsuo K, Yahata T*, et al.* Effective expansion of engrafted human hematopoietic stem cells in bone marrow of mice expressing human Jagged1. *Experimental hematology* 2014 Jun; **42**(6): 487-494 e481.

5. Kawamoto T. Use of a new adhesive film for the preparation of multi-purpose fresh-frozen sections from hard tissues, whole-animals, insects and plants. *Archives of histology and cytology* 2003 May; **66**(2): 123-143.

6. Muguruma Y, Matsushita H, Yahata T, Yumino S, Tanaka Y, Miyachi H*, et al.* Establishment of a xenograft model of human myelodysplastic syndromes. *Haematologica* 2011 Apr; **96**(4): 543-551.

7. Muguruma Y, Yahata T, Miyatake H, Sato T, Uno T, Itoh J*, et al.* Reconstitution of the functional human hematopoietic microenvironment derived from human mesenchymal stem cells in the murine bone marrow compartment. *Blood* 2006 Mar 1; **107**(5):

1878-1887.

8. Houde C, Li Y, Song L, Barton K, Zhang Q, Godwin J*, et al.* Overexpression of the NOTCH ligand JAG2 in malignant plasma cells from multiple myeloma patients and cell lines. *Blood* 2004 Dec 1; **104**(12): 3697-3704.

9. Chiron D, Maiga S, Descamps G, Moreau P, Le Gouill S, Marionneau S*, et al.* Critical role of the NOTCH ligand JAG2 in self-renewal of myeloma cells. *Blood cells, molecules & diseases* 2012 Apr 15; **48**(4): 247-253.

10. Deleu S, Lemaire M, Arts J, Menu E, Van Valckenborgh E, Vande Broek I*, et al.* Bortezomib alone or in combination with the histone deacetylase inhibitor JNJ-26481585: effect on myeloma bone disease in the 5T2MM murine model of myeloma. *Cancer research* 2009 Jul 1; **69**(13): 5307-5311.
