# Supplementary figures and images for: Jagged1-induced Notch activation contributes to the acquisition of bortezomib resistance in myeloma cells
Source: Blood Cancer J. 2017 Dec 15;7(12):650. doi: 10.1038/s41408-017-0001-3 (PMC5802593; doi:10.1038/s41408-017-0001-3)

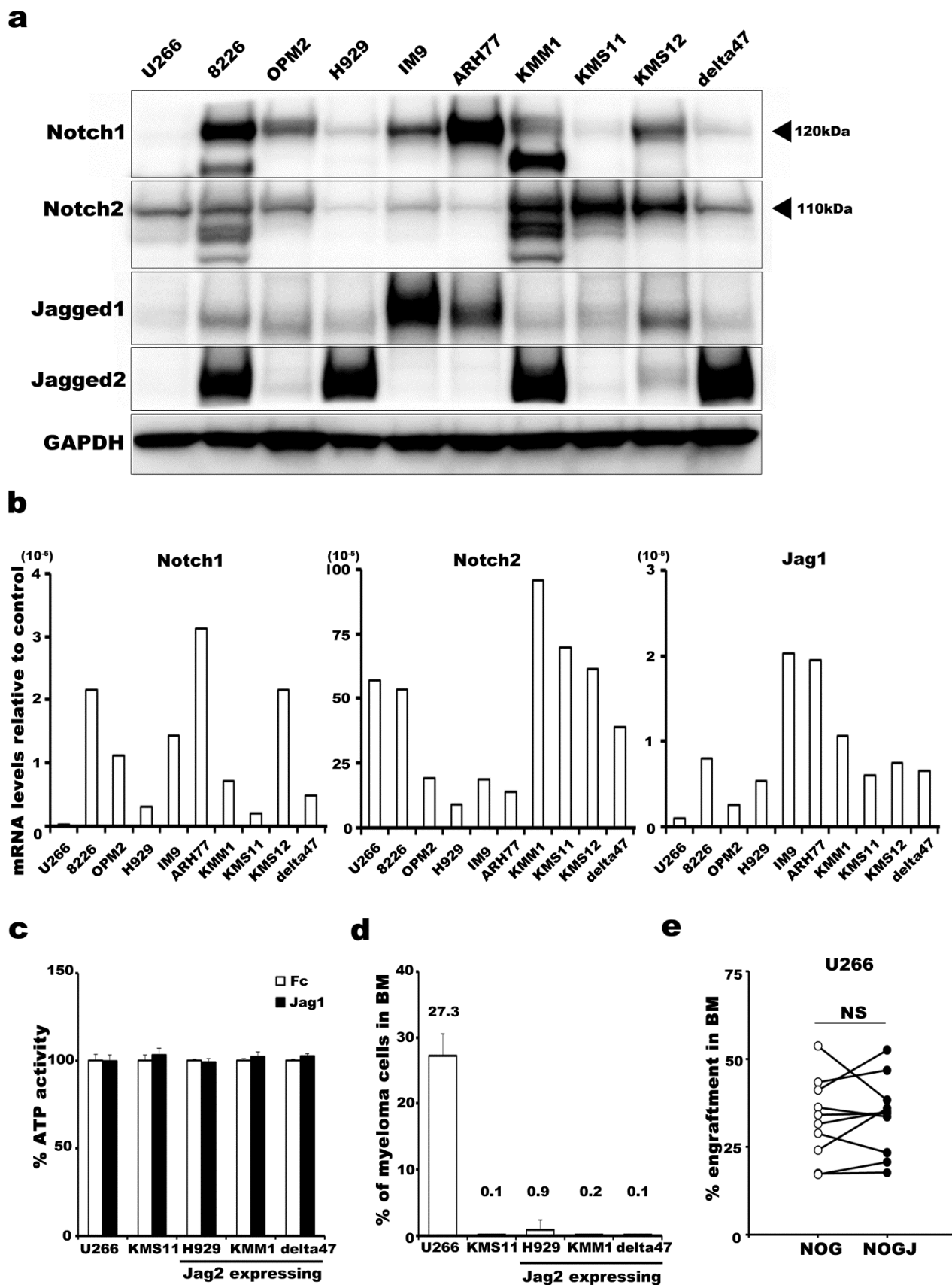

Supplementary Figure1

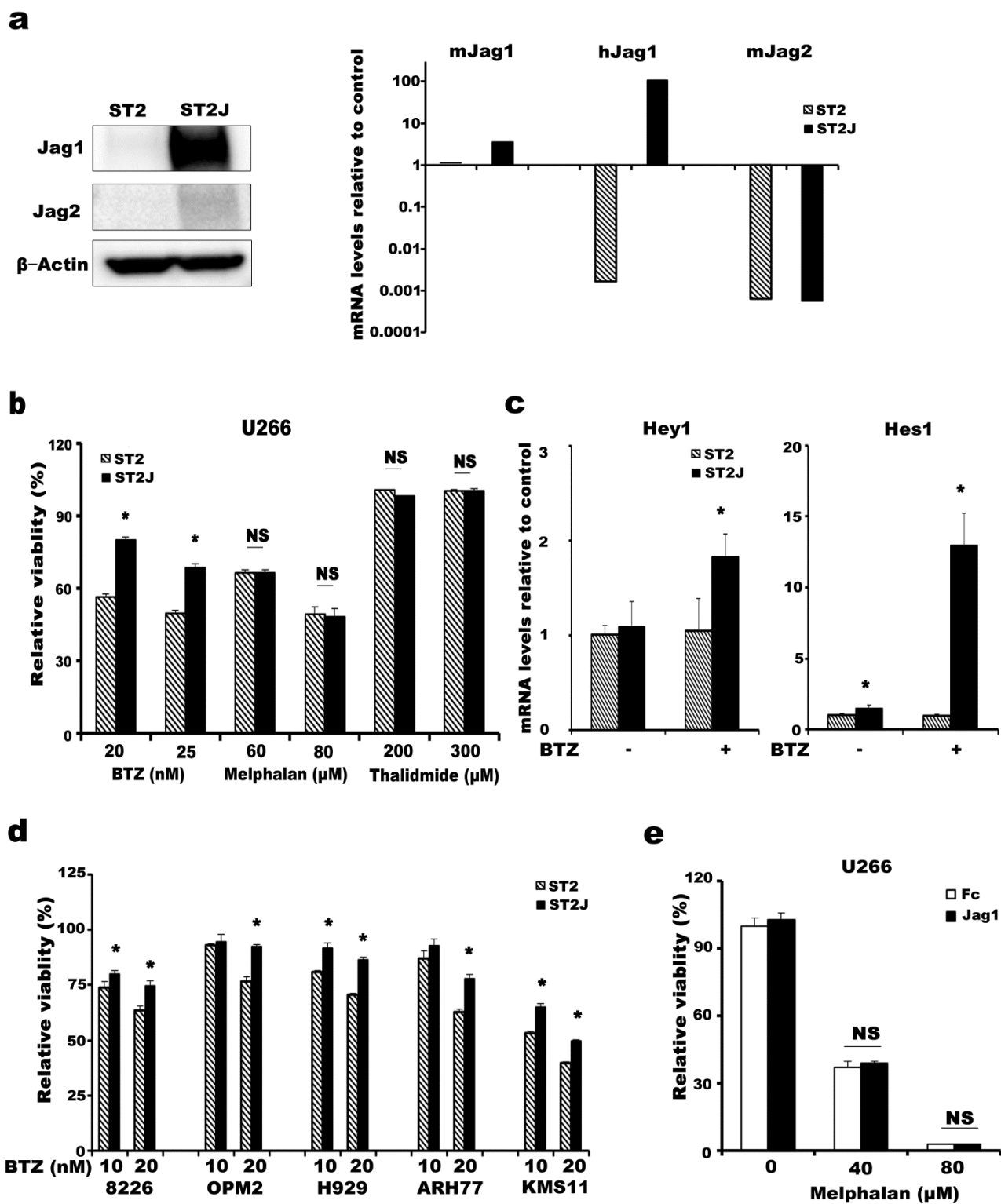

**Supplementary Figure 2**

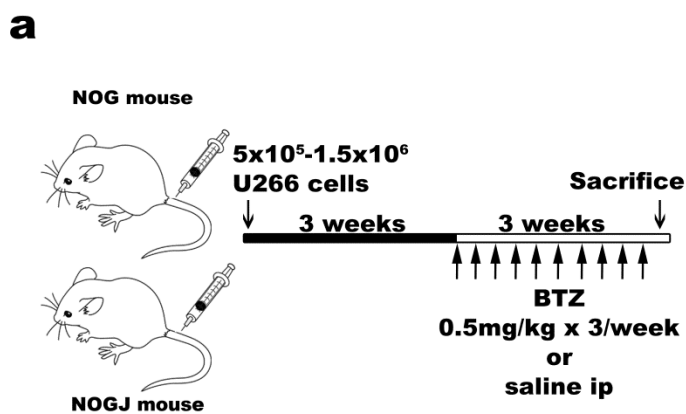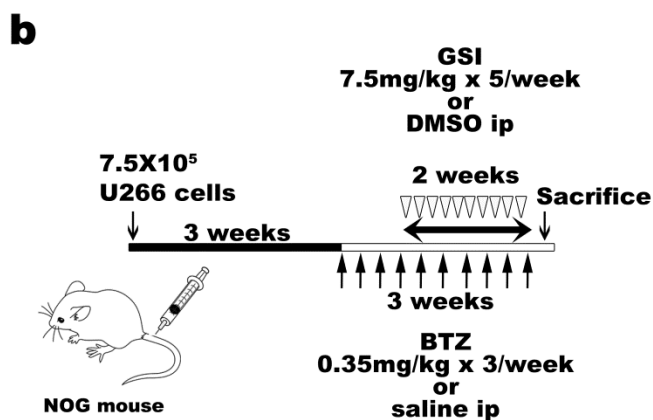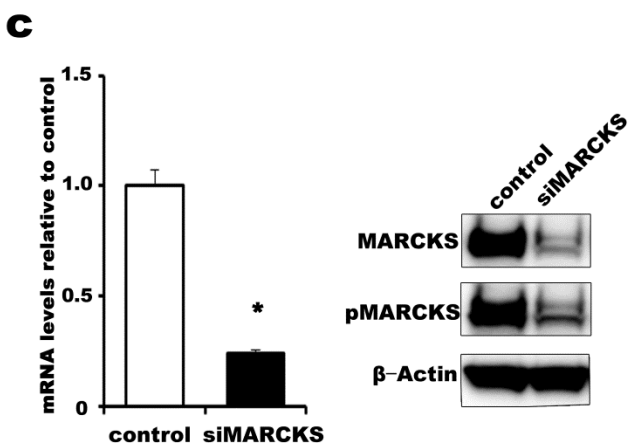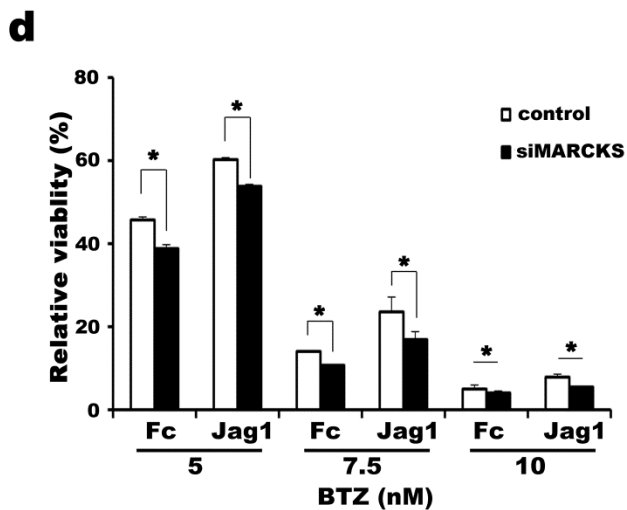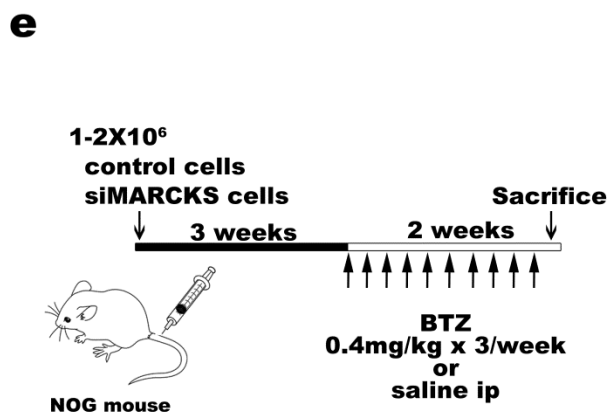

**Supplementary Figure 3**

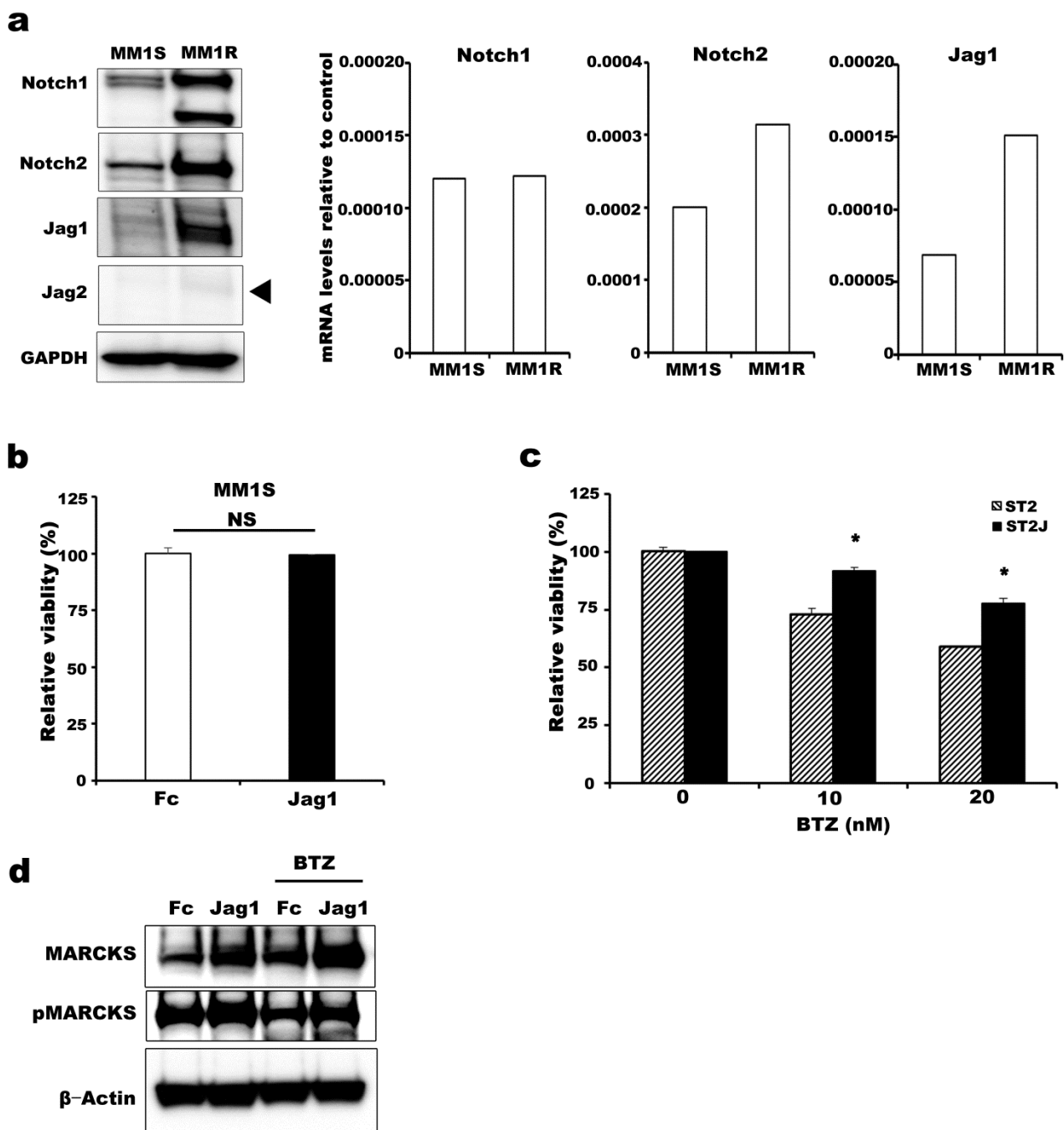

**Supplementary Figure 4**

Supplement: Supplementary file 2 — Supplementary Figures [file 41408_2017_1_MOESM2_ESM.pdf]
